# Anti-neuropathic effects of astaxanthin in a rat model of chronic constriction injury: passing through opioid/benzodiazepine receptors and relevance to its antioxidant and anti-inflammatory effects

**DOI:** 10.3389/fphar.2024.1467788

**Published:** 2024-11-25

**Authors:** Boshra Hashemi, Sajad Fakhri, Amir Kiani, Fatemeh Abbaszadeh, Shahram Miraghaee, Mohammad Mohammadi, Javier Echeverría

**Affiliations:** ^1^ Student Research Committee, Kermanshah University of Medical Sciences, Kermanshah, Iran; ^2^ Pharmaceutical Sciences Research Center, Health Institute, Kermanshah University of Medical Sciences, Kermanshah, Iran; ^3^ Regenerative Medicine Research Center (RMRC), Kermanshah University of Medical Sciences, Kermanshah, Iran; ^4^ Neurobiology Research Center, Shahid Beheshti University of Medical Sciences, Tehran, Iran; ^5^ Medical Biology Research Center, Health Technology Institute, Kermanshah University of Medical Sciences, Kermanshah, Iran; ^6^ Department of Radiology and Nuclear Medicine, School of Paramedical Sciences, Kermanshah University of Medical Sciences, Kermanshah, Iran; ^7^ Departamento de Ciencias del Ambiente, Facultad de Química y Biología, Universidad de Santiago de Chile, Santiago, Chile

**Keywords:** astaxanthin, chronic constriction injury, inflammation, oxidative stress, opioid receptor, benzodiazepine receptor, neuropathic pain

## Abstract

**Introduction:**

Neuropathic pain is a debilitating neurological disorder and is on the rise. Since no effective treatment has been so far approved to combat the complex pathological mechanisms behind neuropathic pain, finding new therapeutic candidates is of great importance. Astaxanthin (AST) is a carotenoid with strong antioxidant, and anti-inflammatory activities.

**Purpose:**

The present research aimed to evaluate the ameliorative effects of AST on a rat model of neuropathic pain.

**Methods:**

To induce neuropathic pain, a chronic constriction injury (CCI) model was employed. Accordingly, Wistar rats were divided into nine groups of six including sham, negative control group (CCI), positive control group gabapentin (100 mg/kg), AST (5, 10 mg/kg), flumazenil (0.5 mg/kg), naloxone (0.1 mg/kg), AST (10 mg/kg) + flumazenil (0.5 mg/kg), and AST (10 mg/kg) + naloxone (0.1 mg/kg) were administered intraperitoneally on days 1, 3, 5, 7, 10, and 14. To check the experimental signs of neuropathic pain and motor dysfunction, hot plate, acetone drop, and open field tests were used at the same time points. Additionally, biochemical assay and zymography were done on days 7 and 14 to assess the changes in catalase, glutathione and nitrite, as well as matrix metalloproteinases (MMP-2 and MMP-9). Besides, histological evaluations were performed for tissue damages on days 7 and 14.

**Results and discussion:**

Results indicated that intraperitoneal injection of AST improved allodynia, hyperalgesia, and locomotor activity after CCI. AST also increased catalase and glutathione while suppressing nitrite, MMP-2, and MMP-9 activity through opioid/benzodiazepine receptors.

**Conclusion:**

The results highlighted AST as a promising candidate against neuropathic pain with beneficial effects on motor function by suppressing inflammatory mediators, and augmenting antioxidant factors, passing through opioid/benzodiazepine receptors.

## 1 Introduction

Neuropathic pain is a debilitating neural disorder following the disruption of neurons’ function and structure, mainly in the somatosensory system (Treede et al., 2008). Neuropathic pain is commonly observed in patients with spinal cord injury, multiple sclerosis, diabetic neuropathy, postherpetic neuralgia, and cancer, which critically reduces life quality (Colloca et al., 2017). Growing studies have been developed to manage neuropathic pain; though, more research is needed to find novel therapeutic agents with higher efficacy and lower side effects (Finnerup et al., 2015).

Chronic constriction injury (CCI) is a model of neuropathic pain, based on nerve damage caused by actual or potential tissue stimuli (Treede et al., 2008). Such pain can be caused by damage to the central or peripheral nervous system, diabetes, metabolic disorders, viral infections, nutritional deficiencies, trauma, and physical injuries (Fonseca-Rodrigues et al., 2021). Neuropathic pain is characterized by signs such as allodynia, hyperalgesia, and dysesthesia (Jensen and Finnerup, 2014). Many drugs have been proposed to control neuropathic pain, including non-steroidal anti-inflammatory drugs (NSAIDs), opiates, tricyclic antidepressants, and anticonvulsant drugs but these drugs suffer from several side effects and have shown low efficacy in the control of neuropathic pain (Muthuraman et al., 2008; Fakhri et al., 2021a). Therefore, many efforts have been made to find a rational and less complicated treatment method for patients with neuropathic pain (Colloca et al., 2017).

Following nerve damage, the signals released from the nerve terminals contribute to microglial activation and propagation of inflammatory cytokines (Soleimani et al., 2019; Zhang et al., 2021) and matrix metalloproteinase 9 (MMP-9) (Fakhri et al., 2021b), which lead to allodynia or hyperalgesia (Kiguchi et al., 2012). Therefore, suppression of inflammatory mediators is believed to relieve neuropathic pain (Kiguchi et al., 2012; Malcangio et al., 2013). On the other hand, oxidative stress factors are also activated following nerve damage; for example, reactive superoxide molecules are increased in the dorsal horn of the spinal cord and dorsal root ganglia as a result of CCI. Also, increased levels of reactive oxygen species (ROS) lead to the initiation and maintenance of central and peripheral sensitization (Bakare and Owoyele, 2020). Previously, the roles of benzodiazepine receptors (O’Connell et al., 2019; Mir et al., 2020; Lape et al., 2023) and opioid receptors (Jones et al., 2023) have been highlighted in pain management.

Astaxanthin (AST), 3,3′-Dihydroxy-beta,beta-carotene-4,4′-dione, is a natural and red-orange keto-carotenoid that belongs to xanthophylls (Fakhri et al., 2018a). Studies have reported that AST possesses significant effects on the control of neuroinflammation- and oxidative stress-mediated neuronal damages (Zarneshan et al., 2020). The findings of another study showed that treatment with AST significantly reduced neuropathic pain by modulating oxidative stress and mitigating astrocytes activation (Sharma et al., 2018). We have also previously shown the potential of AST in combating spinal cord injury-induced neuropathic pain through different mechanisms (Fakhri et al., 2018b; Fakhri et al., 2019).

Therefore, in this study, we sought to investigate the effects of AST on CCI-induced neuropathic pain and motor dysfunction, highlighting its anti-inflammatory and antioxidant capabilities passing through opioid/benzodiazepine receptors.

## 2 Materials and methods

### 2.1 Chemicals and reagents

Astaxanthin (multiple mechanisms; 3,3′-Dihydroxy-β-carotene-4,4′-dione; C_40_H_52_O_4_) and gabapentin (GABA synthetic enzyme modulator; 1-(Aminomethyl)-cyclohexaneacetic acid; C_9_H_17_NO_2_) were purchased from Sigma–Aldrich (Sigma Chemical Co., St. Louis, MO, United States). Naloxone [μ-opioid receptor antagonist; (5α)-4,5-Epoxy-3,14-dihydroxy-17-(2-propen-1-yl) morphinan-6-one; C_19_H_21_NO_4_; Caspian Tamin Pharmaceutical Company, Iran], flumazenil [GABA_A_ receptor antagonist; 5,6-dihydro-4H-imidazo(1,5-a)(1,4)benzodiazepine; C_15_H_14_FN_3_O_3_; Hameln, Germany], ketamine [NMDA receptor antagonist; 2-(2-Chlorophenyl)-2-(methylamino) cyclohexanone, 2-(2-Chlorophenyl)-2-(methylamino)cyclohexanone hydrochloride; Alfasan IBV, Turkey], and xylazine [α2 adrenergic receptor agonist; 2-(2,6-Dimethylphenylamino)-5,6-dihydro-4H-thiazine; C_12_H_16_N_2_S; Alfasan IBV, Turkey] were analytical grade reagents purchased from commercial sources.

### 2.2 Experimental animals

In this research, 54 adult male Wistar rats (weighing 230–250 g) were selected from the reproductive colony of Kermanshah University of Medical Sciences. They were kept in a suitable situation with access to food and water with a 12-h light/dark cycle, a temperature of 25 ± 2°C, and also at optimum humidity. Also, all the protocols were confirmed by the Ethics Committee in Medical Research of Kermanshah University of Medical Sciences (Ethical code: IR.KUM.REC.1400.431). In the current study lowest numbers of animals and surgeries were done in a clean situation (Soleimani et al., 2019; Zhang et al., 2021).

### 2.3 Chronic constriction injury

To evaluate the effect of AST on neuropathic pain, the CCI was induced according to the Bennett method (Bennett and Xie, 1988). For this purpose, rats were anesthetized with 50 mg/kg ketamine and 5 mg/kg xylazine. Following disinfection, incision of muscles and skin, and appearing the sciatic nerve on the left side, four loos chromic catgut (4.0) were tied on the sciatic nerve exactly near the trifurcation of the sciatic. Finally, after neuropathy induction, the animals were transferred to the cage for recovery (Amin et al., 2016; Soleimani et al., 2019).

### 2.4 Experimental groups

In total, 54 rats were divided into nine groups of six, including, the sham group (undergoing surgery without CCI and receiving DMSO 5%), CCI group (undergoing CCI and receiving DMSO 5%), gabapentin (positive control group, 100 mg/kg in DMSO 5%), AST-treated groups (5 and 10 mg/kg in DMSO 5%), flumazenil (FLU) (0.5 mg/kg), naloxone (NAL) (0.1 mg/kg), AST (10 mg/kg) + flumazenil (0.5 mg/kg) (AST + FLU), and AST (10 mg/kg) + naloxone (0.1 mg/kg) (AST + NAL) (Supplementary Figures 1, 2). All groups received intraperitoneal doses on days 1, 3, 5, 7, 10, and 14, about 1 h prior to assessing behavioral tests. Animals were anesthetized on days 7 and 14 with ketamine/xylazine (100/10 mg/kg), and blood samples were obtained from the retro orbital sinus to evaluate the activities of MMP-2 and MMP-9. Besides, glutathione, catalase, and nitrite levels were measured in serum samples. Finally, the sciatic nerve was separated for histopathological analysis (Soleimani et al., 2019). Blood sampling from retro orbital sinus by an expert is a valuable method without animal expiration after sampling. This method allows serial sampling even in different days and researchers can continue performing experiments on the animals until the end of study timeline (e.g., in our study sampling was done on day 7 and rats were studied until day 14 for behavioural analysis and re-sampling). This strategy also minimizes the number of rats as per the National Institutes of Health Guidelines on the care and use of laboratory animals. Once required blood sampling is done, the capillary tube is removed and wiped with sterile cotton. Bleeding can be stopped using gentle finger pressure. Then, rats should be housed individually in large cages and monitored, until the possible bleeding is stopped. *Ad libitum* intake of water helps replace the lost blood (Sharma et al., 2014; Fakhri et al., 2021b).

### 2.5 Behavioral tests

Behavioral tests were done on days 1, 3, 5, 7, 10, and 14 after surgery. Open field tests were used for the evaluation of motor activity. In addition, acetone drop and hot plate tests were done to assess cold allodynia and heat hyperalgesia, respectively. All behavioral tests were carried out as per the mentioned order at 30-min intervals.

#### 2.5.1 Acetone drop test

The acetone drop test was used to evaluate cold allodynia. To ensure proper access to the paws, rats were placed on a metal mesh grid during the test. To examine the response to cold stimulation of the hind paw, 100 μL acetone was sprayed on the plantar surface of the paw for 2–3 s from a distance of 2 cm. Response to cold was classified with the following scales: 0: no response; 1: shocking response without paw withdrawal; 2: brief retraction of paw; 3: long-term withdrawal of the paw that lasts between 5–30 s and is often accompanied by flinching and licking the paw; 4: prolonged and frequent withdrawal (30 s) accompanied by shaking, licking and/or vocalization (Kauppila, 2000; Fakhri et al., 2018b).

#### 2.5.2 Hot plate test

In this experiment, we assessed thermal hyperalgesia related to neuropathic pain in rats by measuring their paw withdrawal latency (PWL) using a hot plate apparatus (Harvard Apparatus, Holliston, MA). Briefly, rats were placed within a clear Plexiglas observation chamber on a hot plate analgesia meter. PWL was defined as the time that it took the rat to exhibit pain-related behaviors, such as paw licking, hindlimb withdrawal, or jumping, obtained from three repeats of the experiment (5 min intervals) after being placed on the hot plate surface at 52 ± 0.5°C. A 60-s cut-off time was applied to each animal to prevent damage to the paw tissue (Bakare and Owoyele, 2020; Fakhri et al., 2022).

#### 2.5.3 Open field test

The open field test was conducted to evaluate the overall motor activity of the animals. Briefly, rats were placed individually in an open field (60 × 60 × 40 cm) with a non-slippery surface. Total distance and velocity were video-recorded in 5 min. The total number of squares crossed by each rat (total crossing), the number of rearing (standing on two hind paws without touching the walls), and grooming were recorded. This experiment was performed in a completely quiet environment with a constant temperature and uniform brightness (Parvardeh and Hosseinzadeh, 2003; Parvardeh et al., 2016). After finalizing each experiment, the entire field was cleaned with ethanol 70%.

### 2.6 Biochemical analysis

To evaluate serum antioxidant potential, catalase and glutathione activities were measured on days 7 and 14. Serum levels of nitrite were also measured to assess oxidative capacity.

#### 2.6.1 Catalase assay

The activity of catalase was measured according to the Aebi method (Aebi, 1984). Since catalase can convert hydrogen peroxide (H_2_O_2_) into water and oxygen, to determine the serum level of catalase, we examined changes in the serum levels of H_2_O_2_. To do this, first 20 μL of serum sample and 100 μL of 65 mM hydrogen peroxide were added to the wells and then incubated for 4 min at 25°C. The reaction was stopped by adding 100 μL of ammonium molybdate at a concentration of 32.4 mM. Finally, the concentration of yellow molybdate complex and hydrogen peroxide was measured at 405 nm by an ELISA reader.

#### 2.6.2 Glutathione assay

In this test, to assess glutathione, the Ellman method was used. This method is based on the glutathione decline by the Ellman’s reagent [5,5′-dithio-bis (2-nitrobenzoic acid) (DTNB)] which leads to the formation of oxidized glutathione-TNB adduct and TNB chromophore. As the rate of DTNB formation is proportional to the amount of glutathione present in the sample, we can estimate the level of plasma glutathione by measuring the amount of TNB formation through spectrophotometry at a wavelength of 412 nm (Tipple and Rogers, 2012; Fakhri et al., 2022). To this aim, 60 µL of serum was mixed with 100 µL of DTNB at a concentration of 2 mg/mL in the wells of a 96-well plate and then 50 µL of phosphate buffer was added and the plate was incubated for 10 min at 37°C. Then, absorbance was read at 412 nm.

Using the following formula, differences of absorbance (%) between sample groups and the sham group was reported in both the glutathione and catalase assays:

$$\left.\left\{\left(C\;sham-C\;sample\right)\right|C\;sham)\right\}\times 100$$

#### 2.6.3 Nitrite assay

The Griess method was employed to measure nitrite levels in the serum (Sun et al., 2003). The serum samples (100 µL) were combined with a sulfanilamide solution (dissolved in 5% HCl) (50 µL) in the plate wells and left in darkness for 5 min. Subsequently, 50 µL of naphthyl ethylene diamine dihydrochloride (NEDD; 0.1% in distilled water) was added, and the plate was incubated in darkness at 37°C for 30 min. The optical density (OD) was then measured at 540 nm using a plate reader. Simultaneously, a standard curve was generated using 100 µL of sodium nitrite at varying concentrations.

### 2.7 Gelatin zymography

The gelatin zymography method was used to evaluate the pattern of gelatinases in the serum. Briefly, serum samples with a total protein content of 100 μg using the Bradford method were loaded on sodium dodecyl sulfate-polyacrylamide gels copolymerized with 0.1% gelatin. Then, electrophoresis was performed at a constant voltage of 150 V using a mini-gel slab device Mini Protean III (Bio-Rad, Hercules, CA). The gel was washed in the renaturation buffer for 1 h on a shaker. The renaturation buffer contained 2.5% Triton X-100 (in 50 mM Tris-HCl). After that, the gel was incubated at 37°C in the incubation buffer for 18 h. The incubation buffer comprises 0.02% NaN_3_, 10 mM CaCl_2_, and 0.15 NaCl in Tris-HCl (50 mM). Respectively, Gel staining was done with Coomassie blue, followed by destaining in acetic acid (5%) and methanol (7%). The formed clear bands indicate gelatinolytic activity. ImageJ software (National Institute of Health, United States) was used to quantify the relative intensity of the bands in compare to the sham group (Fakhri et al., 2021b; Fakhri et al., 2022).

### 2.8 Histological analysis

In this research, rats were anesthetized and sacrificed with a mixture of intraperitoneal xylazine (20 mg/kg) and ketamine (100 mg/kg). Samples from each group were selected on the 7th and 14th day. The close parts of the ligated sciatic nerves were excised and fixed with a 10% formalin solution (Muthuraman et al., 2008). After that, tissues were kept in paraffin. Using hematoxylin and eosin (H&E), consecutive sections of the injury site were stained (Sudoh et al., 2004), and then the sections were qualitatively examined by experts to investigate axonal degeneration under a light microscope (×400) in accordance with routine histological techniques.

### 2.9 Statistical analysis

The obtained results were assessed with Prism version 8.0 software and a Two-way ANOVA statistical test and Bonferroni *post hoc* for behavioral analysis. One-way ANOVA statistical test with Tukey *post hoc* test were employed for biochemical analysis. The area under the curve (AUC) was calculated using the linear trapezoidal method. All the data are presented as mean ± SEM and significant level was set at *p* < 0.05.

## 3 Results

### 3.1 Acetone drop test

**FIGURE 1:**
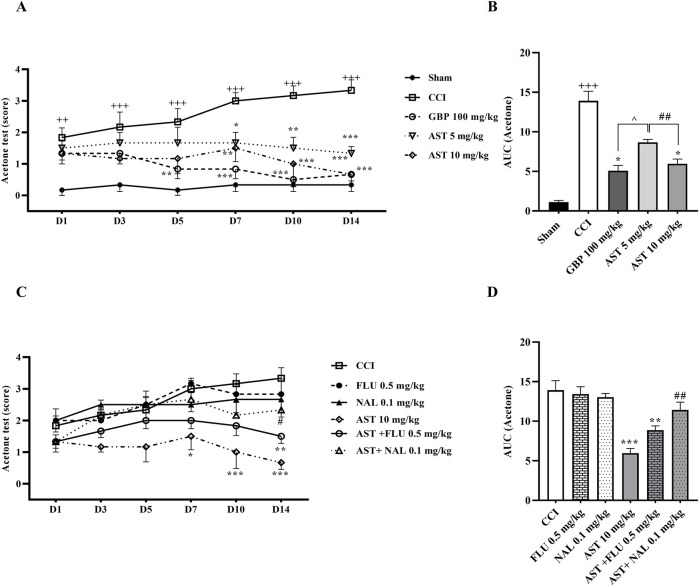
Effects of astaxanthin on cold allodynia and its mechanisms after CCI in rats. CCI significantly decreased tolerance threshold of cold stimulus that was reversed by AST **(A)**, and confirmed by AUC analysis **(B)**. While FLU and NAL alone did not affect cold allodynia, those administrations with AST partially reduced AST’s effects **(C)**, and the result was confirmed by AUC analysis **(D)**. The data are shown as mean ± SEM (*n* = 6). ^++^ *p* < 0.01, ^+++^ *p* < 0.001 vs. sham. *p < 0.05, **p < 0.01, ***p < 0.001 vs. CCI, ^##^ *p* < 0.001 vs. AST 10, ^^^ *p* < 0.05 vs. GBP. CCI, chronic constriction injury; GBP, Gabapentin; AST, Astaxanthin; FLU, Flumazenil; NAL, Naloxone.

The results of the acetone drop test indicated that the paw withdrawal reflex in the CCI group had a considerable increase compared to the sham group (*p* < 0.01) (Figure 1A). Moreover, the results indicated that treatment with AST in both doses increased the tolerance threshold of cold stimulus after CCI, with the better result seen in the group receiving AST at the dose 10 mg/kg. This group showed a remarkable decrease in cold allodynia compared to the CCI group on days 7 (*p* < 0.01), 10 and 14 (*p* < 0.001) (Figure 1A). Also, in this group the area under the curve of cold allodynia shad a remarkable decrease compared to the CCI group (*p* < 0.05) (Figure 1B).

On the other hand, the paw withdrawal reflex in response to the cold stimulus in the rats receiving flumazenil and naloxone alone was not considerably different from the CCI group (Figure 1C). Nevertheless, the results showed that the intraperitoneal injection of naloxone half an hour before AST (10 mg/kg) remarkably reduced the analgesic activity of AST on day 14 (*p* < 0.01), which led to an increase in cold allodynia in this group (Figure 1C). In addition, in this group, the area under the curve of cold allodynia also indicated a considerable increase compared to the group treated with AST (10 mg/kg) (*p* < 0.01) (Figure 1D).

### 3.2 Hot plate test

**FIGURE 2:**
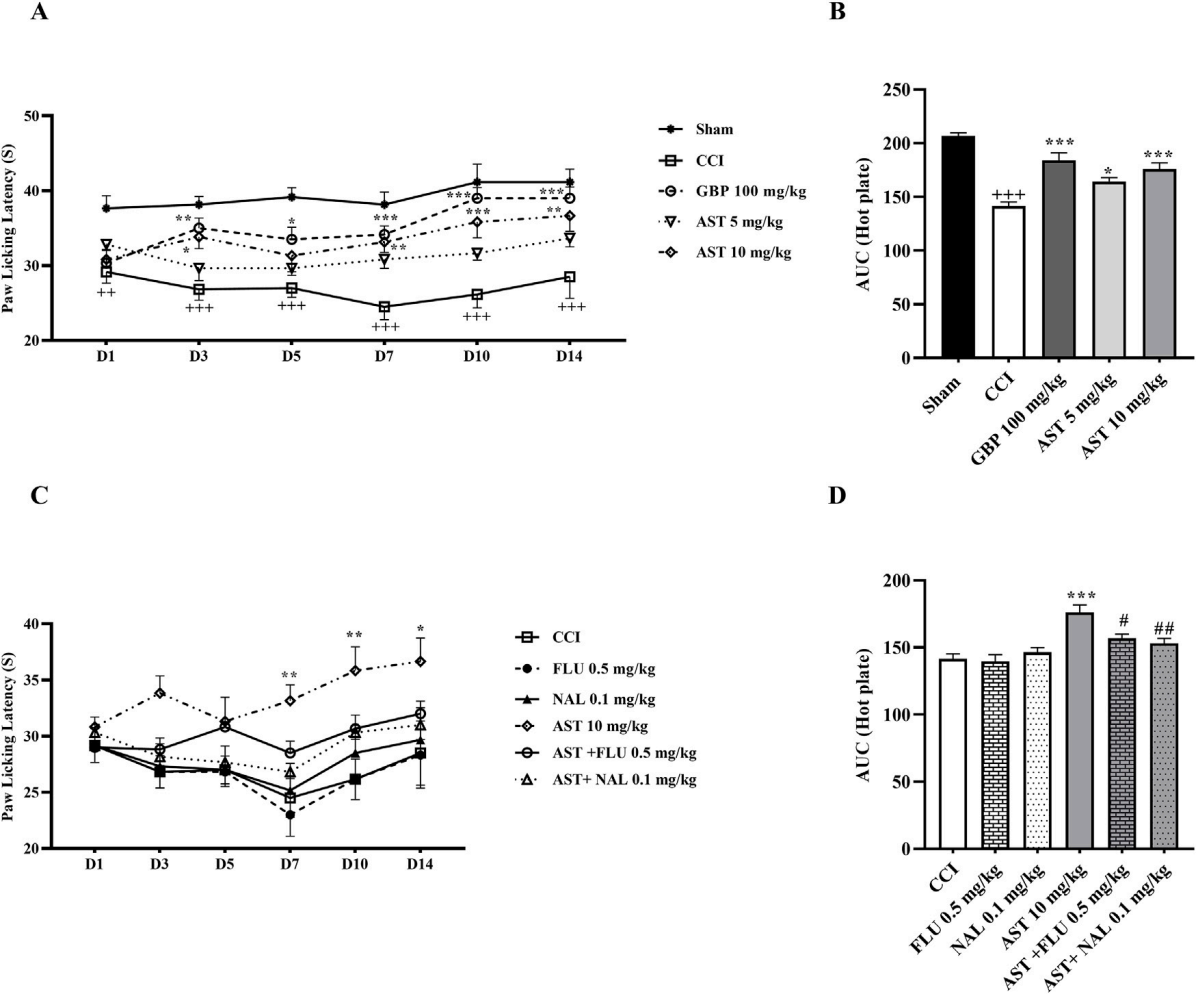
Effects of astaxanthin on thermal hyperalgesia and its mechanisms after CCI in rats. CCI significantly decreased paw licking latency during thermal hyperalgesia, that was reversed by AST **(A)**. The result was confirmed by AUC analysis **(B)**. While FLU and NAL alone did not affect thermal hyperalgesia, those administrations with AST partially reduced AST’s effect **(C)**, and was confirmed by AUC analysis **(D)**. The data are shown as mean ± SEM (*n* = 6). ^++^ *p* < 0.01, ^+++^ *p* < 0.001 vs. sham. *p < 0.05, **p < 0.01, ***p < 0.001 vs. CCI, ^#^ *p* < 0.05^, ##^ *p* < 0.01 vs. AST 10. CCI, chronic constriction injury; GBP, Gabapentin; AST, Astaxanthin; FLU, Flumazenil; NAL, Naloxone.

The data of the hot plate test (Figure 2A) indicated that the time delay of paw withdrawal (PWL) by rats, as the time to reach the pain threshold by heat, was the same in the sham group animals and they were unresponsive. In the CCI group, rats strongly reacted to heat and exhibited a considerable decline in PWL time compared to the first group. The significant differences were seen on the first day with *p* < 0.01 and from days 3–14 with *p* < 0.001. Likewise, the data indicated that treatment with AST caused an increase in PWL delay following CCI, and the better result was related to the group receiving AST at the dose of 10 mg/kg. PWL delay showed a considerable increase in this group compared to the CCI group on days 7 and 14 (*p* < 0.01) and on day 10 (*p* < 0.001) (Figure 2A). In addition, the area under the PWL curve in the groups treated with AST was significantly increased compared to the CCI group, and this increase was observed in AST 5 mg/kg (*p* < 0.05) and AST 10 mg/kg (*p* < 0.001) groups (Figure 2B). In this study, in rats receiving flumazenil and naloxone, PWL delay did not differ compared to the CCI group (Figure 2C). Besides, the data showed that the area under the PWL curve in the AST + FLU (*p* < 0.05) and AST + NAL (*p* < 0.01) was significantly decreased compared to the AST group (10 mg/kg) indicating a significant increase in thermal hyperalgesia after blocking the opioid and benzodiazepine receptors (Figure 2D).

### 3.3 Open field test

**FIGURE 3:**
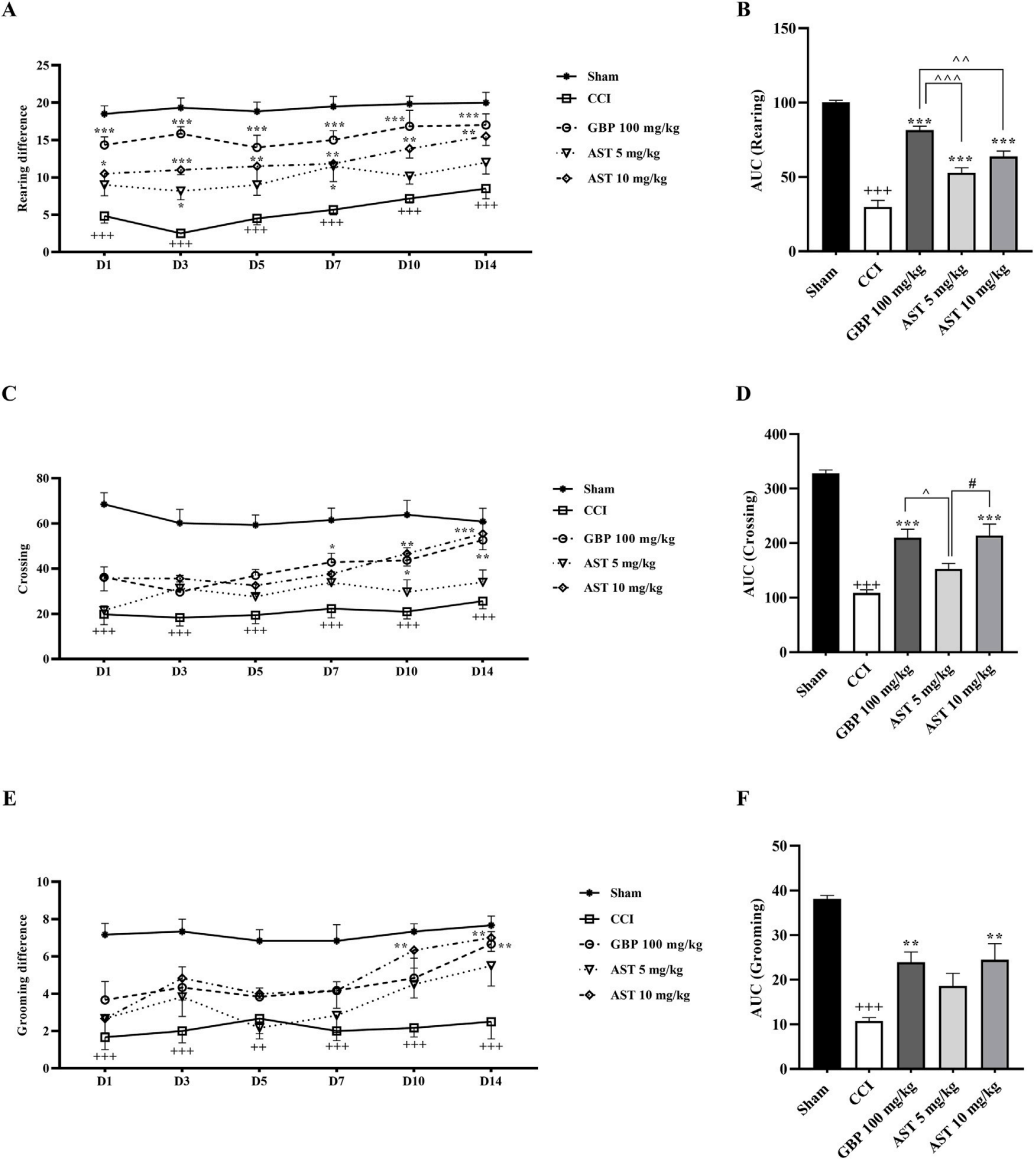
Effects of astaxanthin on locomotor activity after CCI in rats. CCI significantly decreased locomotor activity during rearing **(A)**, crossing **(C)** and grooming **(E)** evaluations, which was reversed by AST 10 mg/kg. The AUC analysis also confirmed the effect of AST on rearing **(B)**, crossing **(D)** and grooming **(F)**. The data are shown as mean ± SEM (*n* = 6). ^+++^ *p* < 0.001 vs. sham, **p < 0.01, ***p < 0.001, vs. CCI, ^^^ *p* < 0.05, ^^^^ *p* < 0.01, ^^^^^ *p* < 0.001, vs. GBP, ^#^ *p* < 0.05 vs. AST 10, CCI, chronic constriction injury; GBP, Gabapentin; AST, Astaxanthin; FLU, Flumazenil; NAL, Naloxone.

The data of the open field test showed that all the three components of locomotor activity were the same in the sham group animals and they were unresponsive (Figure 3). The numbers of rearing (Figure 3A), crossing (Figure 3C) and grooming (Figure 3E) in animals of the CCI group were greatly decreased compared to the sham group (*p* < 0.001).

Results also showed that AST improved all the three motor function parameters compared to the CCI group (Figure 3) (*p* < 0.01). The area under the curve also illustrated the reduction of these three movement parameters following the CCI model (*p* < 0.001), along with the beneficial effect of AST in those parameters (Figures 3B, D, F) (*p* < 0.01).

**FIGURE 4:**
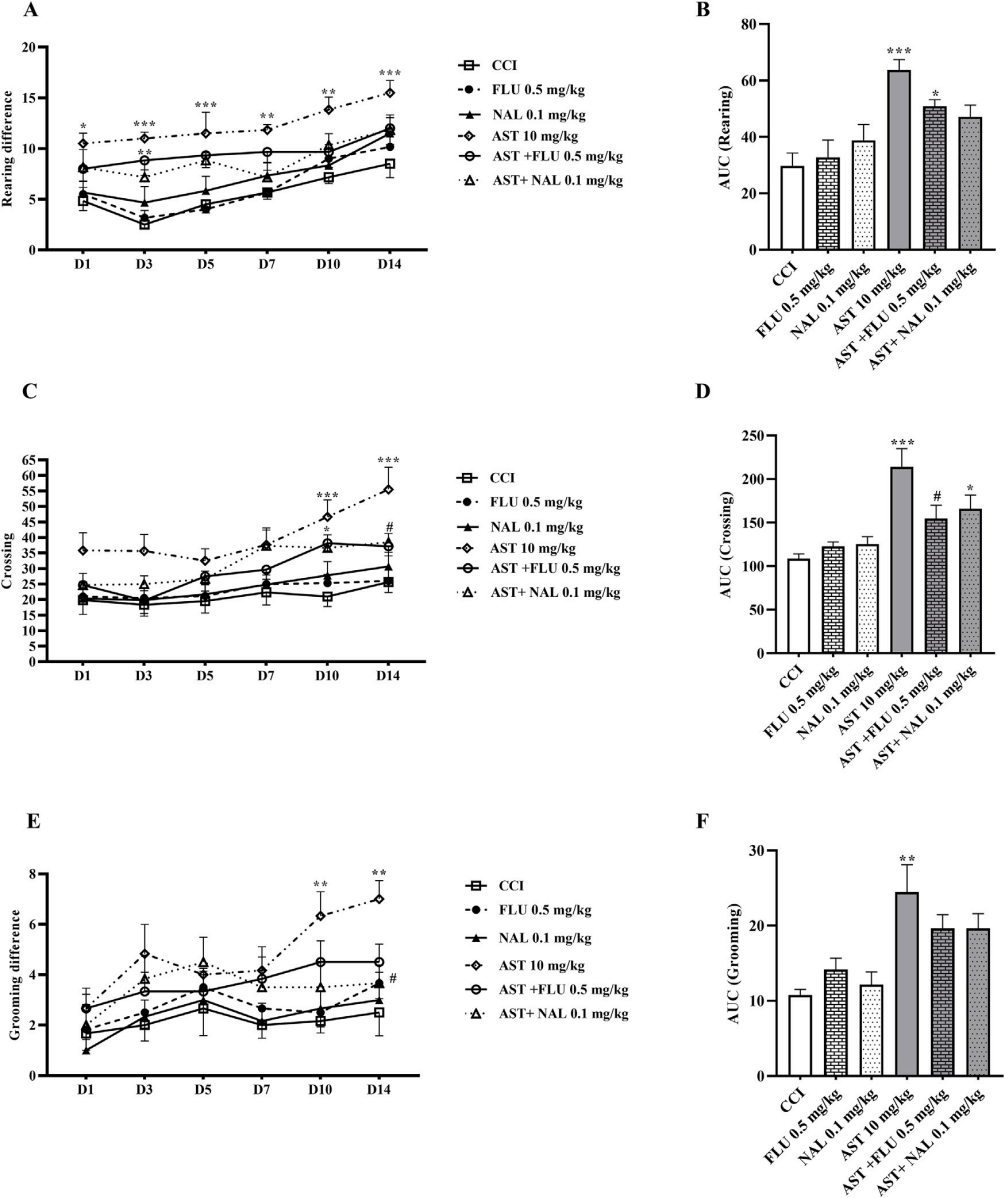
Effects of astaxanthin co-administered with FLU and NAL on locomotor activity after CCI in rats. FLU and NAL alone did not affect locomotor activity during rearing **(A)**, crossing **(C)** and grooming **(E)**, but they reduced AST’s effect. AUC analysis confirmed the effect of NAL and FLU on rearing **(B)**, crossing **(D)** and grooming **(F)**. The data are shown as mean ± SEM (*n* = 6). *p < 0.05, **p < 0.01, ***p < 0.001 vs. CCI, ^#^ *p* < 0.05 vs. AST 10. CCI, chronic constriction injury; GBP, Gabapentin; AST, Astaxanthin; FLU, Flumazenil; NAL, Naloxone.

In the groups receiving flumazenil and naloxone alone, the locomotor activity of rats in all the three components including rearing (Figure 4A), crossing (Figure 4C) and grooming (Figure 4E) did not show any remarkable difference compared to those in the CCI group. However, these parameters were partially reduced in the groups receiving AST + NAL and AST + FLU compared to the AST group (10 mg/kg), which was significant in crossing (*p* < 0.05). The area under the curve clearly showed such changes (Figures 4B, D, F).

In addition to the aforementioned Figures, a Supplementary Figure also provided to refuse the role of solvent volume on neuropathic pain and motor dysfunction (Sham, sham received v2, CCI, CCI received v2) (Supplementary Figures 1, 2). Since unnecessarily use of different rats is against the National Institutes of Health Guidelines on the care and use of laboratory animals nothing role of solvent volume extrapolated to biochemical and histological analysis.

### 3.4 Glutathione assay

**FIGURE 5:**
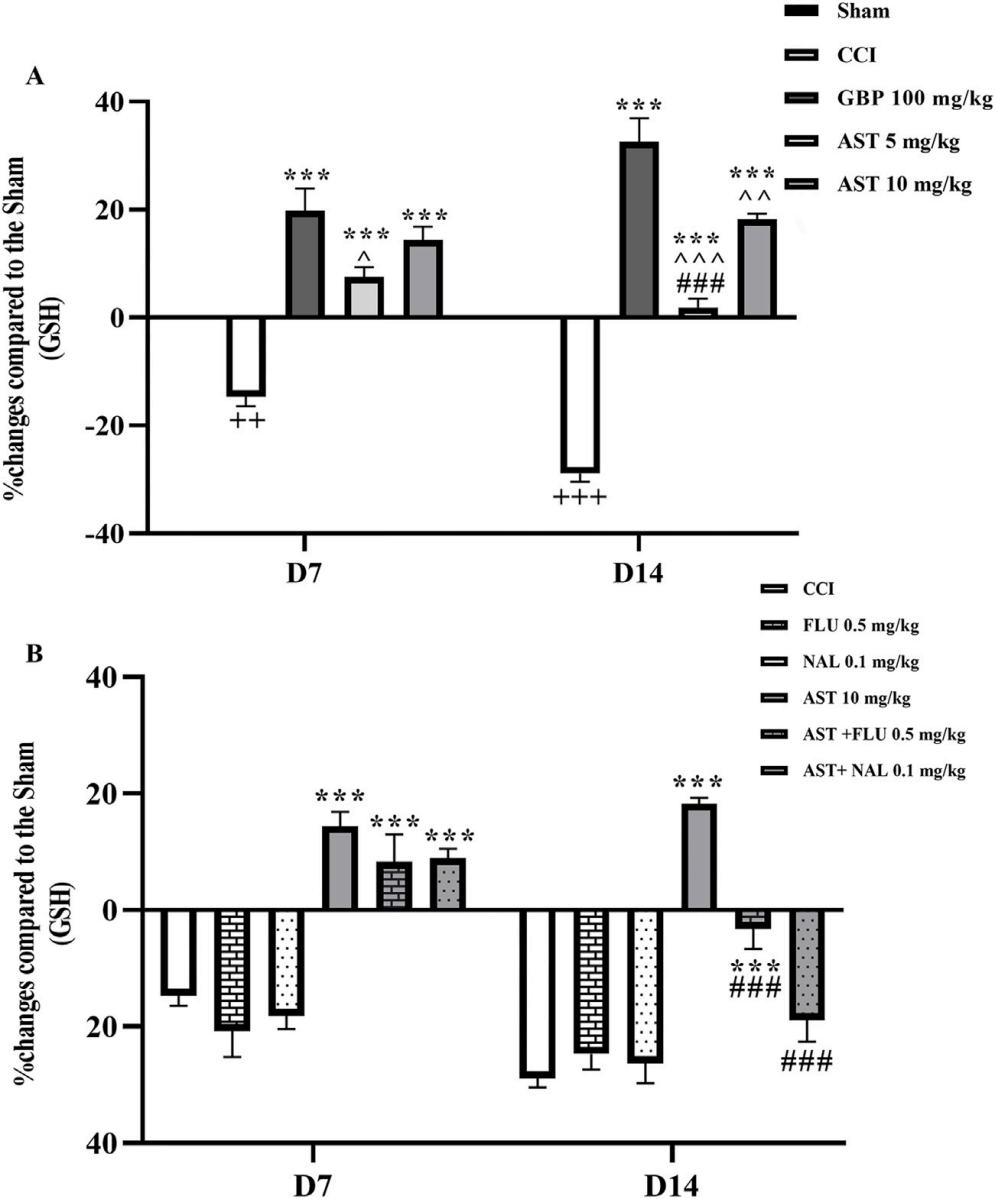
Effects of astaxanthin on serum glutathione level and its mechanisms after CCI in rats. CCI significantly decreased glutathione level, which was reversed by AST 10 mg/kg **(A)**. While FLU and NAL alone did not affect serum glutathione level, those administrations with AST partially reduced AST’s effects **(B)**. The data are shown as mean ± SEM (*n* = 3). ^++^ *p* < 0.01, ^+++^ *p* < 0.001 vs. sham, ***p < 0.001, vs. CCI, ^^^ *p* < 0.05, ^^^^ *p* < 0.01, ^^^^^ *p* < 0.001, vs. GBP, ^###^ *p* < 0.001 vs. AST 10. CCI, chronic constriction injury; GBP, Gabapentin; AST, Astaxanthin; FLU, Flumazenil; NAL, Naloxone.

The serum level of glutathione in CCI group compared to the sham group considerably declined on days 7 and 14, which was related to the antioxidant activity of glutathione (*p* < 0.01) (Figure 5A). The intraperitoneal injection of AST at both doses, especially the dose of 10 mg/kg increased the level of glutathione compared to the CCI group (*p* < 0.001) (Figure 5A). Administration of naloxone and flumazenil alone did not change serum glutathione levels as compared to the CCI group. However, in both AST + NAL and AST + FLU groups, serum levels of glutathione were shown to be significantly decreased compared to the AST group (10 mg/kg) (*p* < 0.001) (Figure 5B).

### 3.5 Catalase assay

**FIGURE 6:**
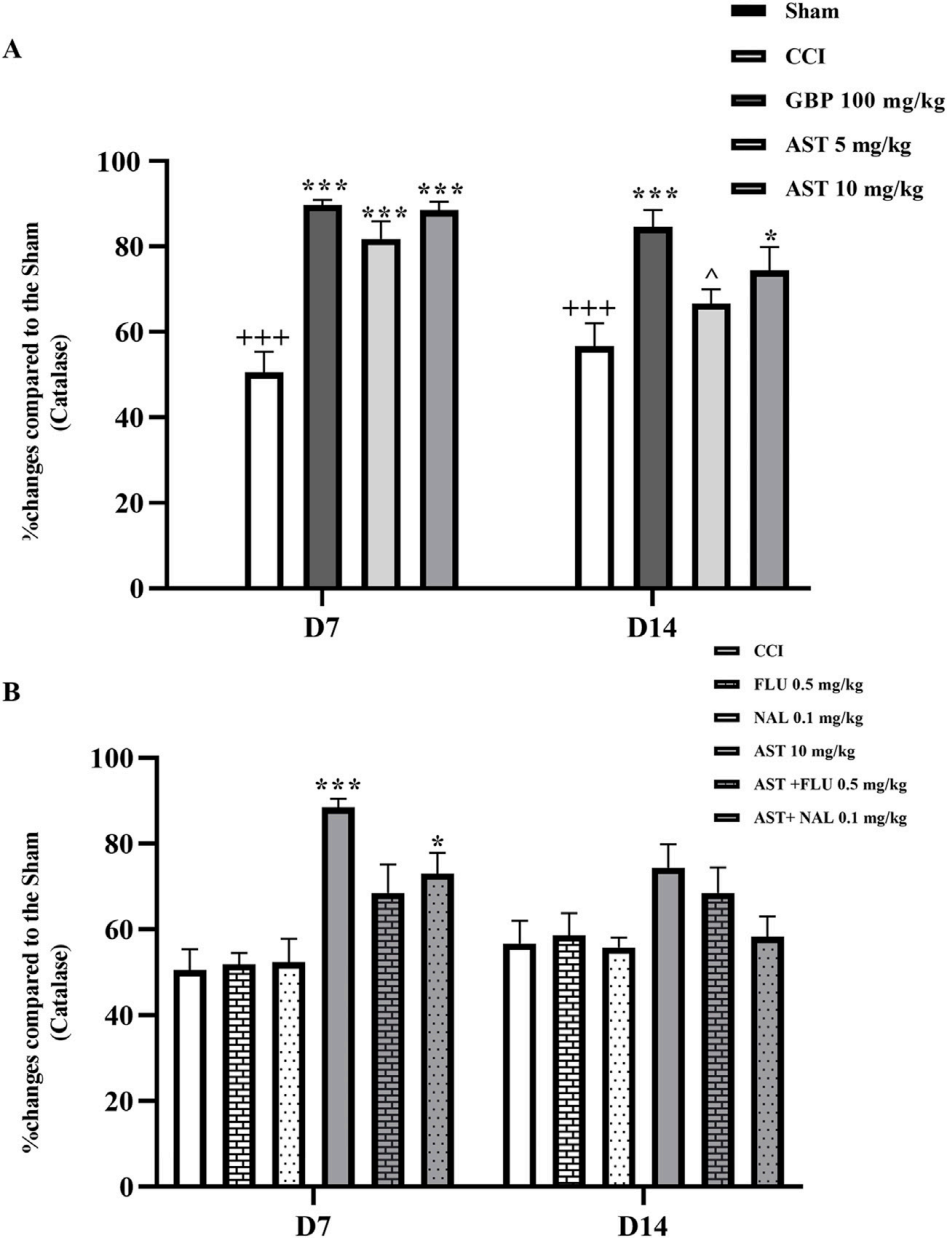
Effects of astaxanthin on the serum level of catalase and its mechanisms after CCI in rats. CCI significantly decreased catalase level, which was reversed by AST 10 mg/kg **(A)**. While FLU and NAL alone did not affect serum catalase level, those administrations with AST partially reduced AST’s effects **(B)**. The data are shown as mean ± SEM (*n* = 3). ^+++^ *p* < 0.001 vs. sham, *p < 0.05, ***p < 0.001 vs. CCI, ^^^ *p* < 0.05 vs. GBP. CCI, chronic constriction injury, GBP, Gabapentin, AST, Astaxanthin, FLU, Flumazenil, NAL, Naloxone.

The data showed (Figure 6A) that the serum level of catalase in the CCI group was significantly decreased compared to the sham group (*p* < 0.001). Besides, the serum levels of catalase at both doses of AST, especially the dose of 10 mg/kg (*p* < 0.05), increased significantly compared to the CCI group. Injection of flumazenil and naloxone alone did not affect the serum levels of catalase in rats. The results of flumazenil and naloxone injections on catalase serum levels were similar to the CCI group. The injection of these two together with AST prevented the positive effect of AST on catalase level (*p* < 0.05) (Figure 6B).

### 3.6 Nitrite assay

**FIGURE 7:**
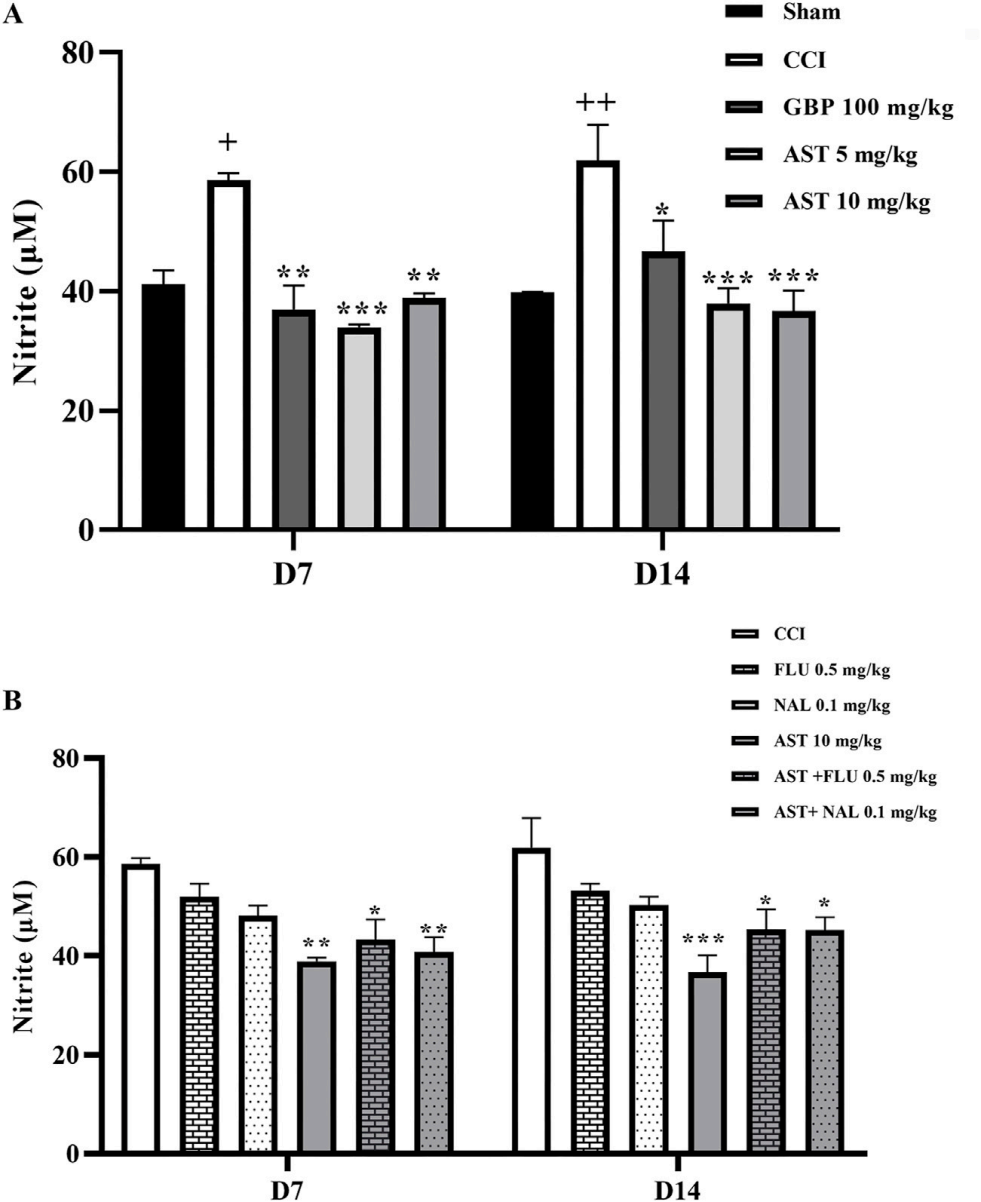
Effects of astaxanthin on serum level of nitrite and its mechanisms after CCI in rats. CCI significantly increased nitrite level, which was reversed by AST 10 mg/kg **(A)**. While FLU and NAL alone did not affect serum nitrite level, those administrations with AST partially reduced AST’s effects **(B)**. The data are shown as mean ± SEM (*n* = 3). ^+^ *p* < 0.05, ^++^ *p* < 0.01 vs. sham, *p < 0.05, **p < 0.01, ***p < 0.001 vs. CCI. CCI, chronic constriction injury; GBP, Gabapentin; AST, Astaxanthin; FLU, Flumazenil; NAL, Naloxone.

Our findings demonstrated a significant increase in serum nitrite levels in the CCI group compared to the sham group (*p* < 0.05). In addition, AST administration was shown to reduce this increased pattern of nitrite following CCI similar to the positive control group (GBP group) (*p* < 0.001) (Figure 7A). The results of flumazenil and naloxone injections on nitrite serum levels were similar to the CCI group. Their injection with AST did not cause considerable changes in the serum levels of nitrite when compared to the group receiving 10 mg/kg AST on days 7 and 14 (Figure 7B).

### 3.7 Gelatin zymography

**FIGURE 8:**
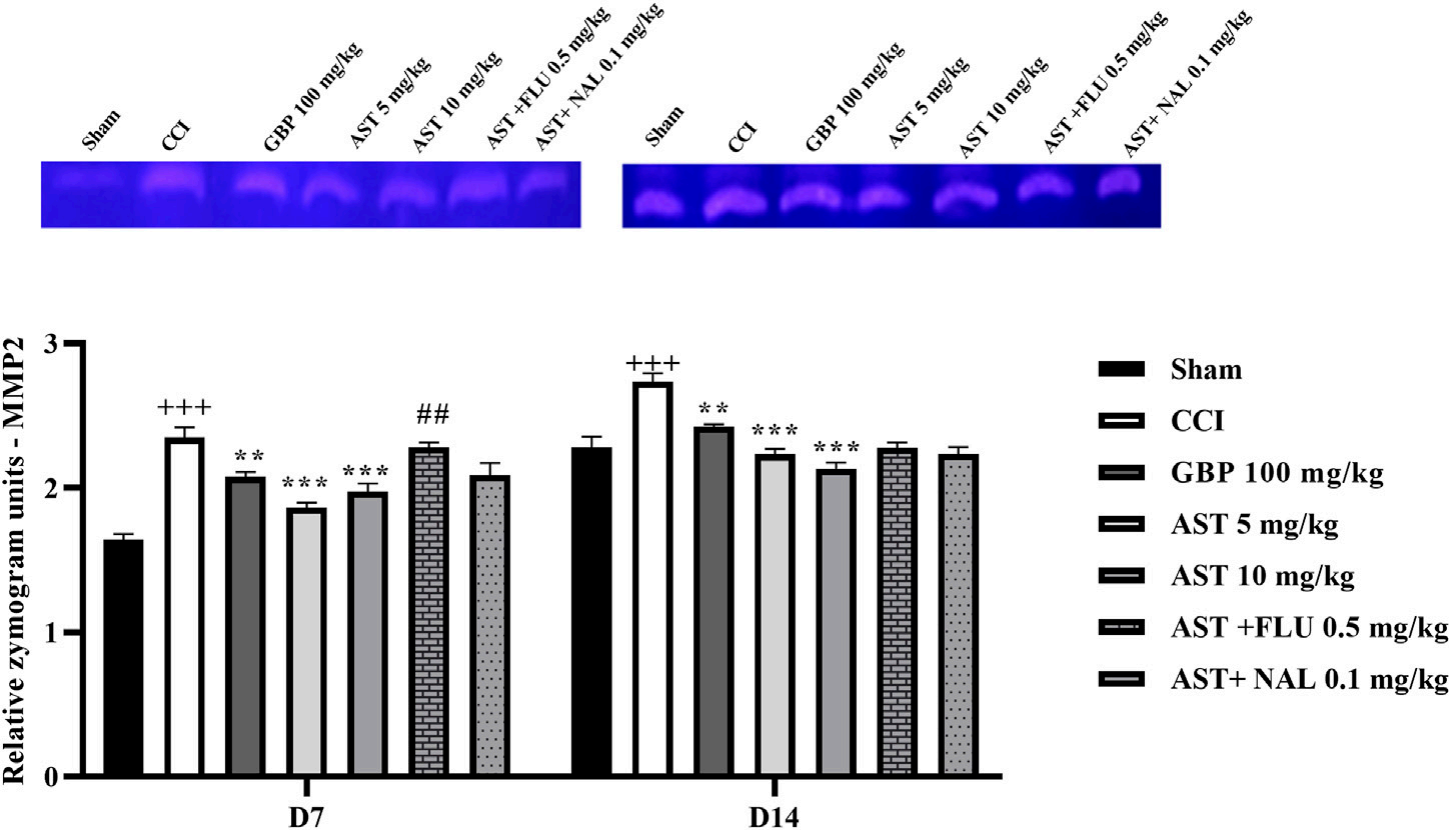
Effects of astaxanthin on serum MMP-2 activity after CCI in rats. The data are shown as mean ± SEM (*n* = 3). ^+++^ *p* < 0.01 vs. sham, **p < 0.01, ***p < 0.001 vs. CCI, ^##^ *p* < 0.01 vs. AST 10. CCI, chronic constriction injury; GBP, Gabapentin; AST, Astaxanthin; FLU, Flumazenil; MMP, Matrix metalloproteinase; NAL, Naloxone.

**FIGURE 9:**
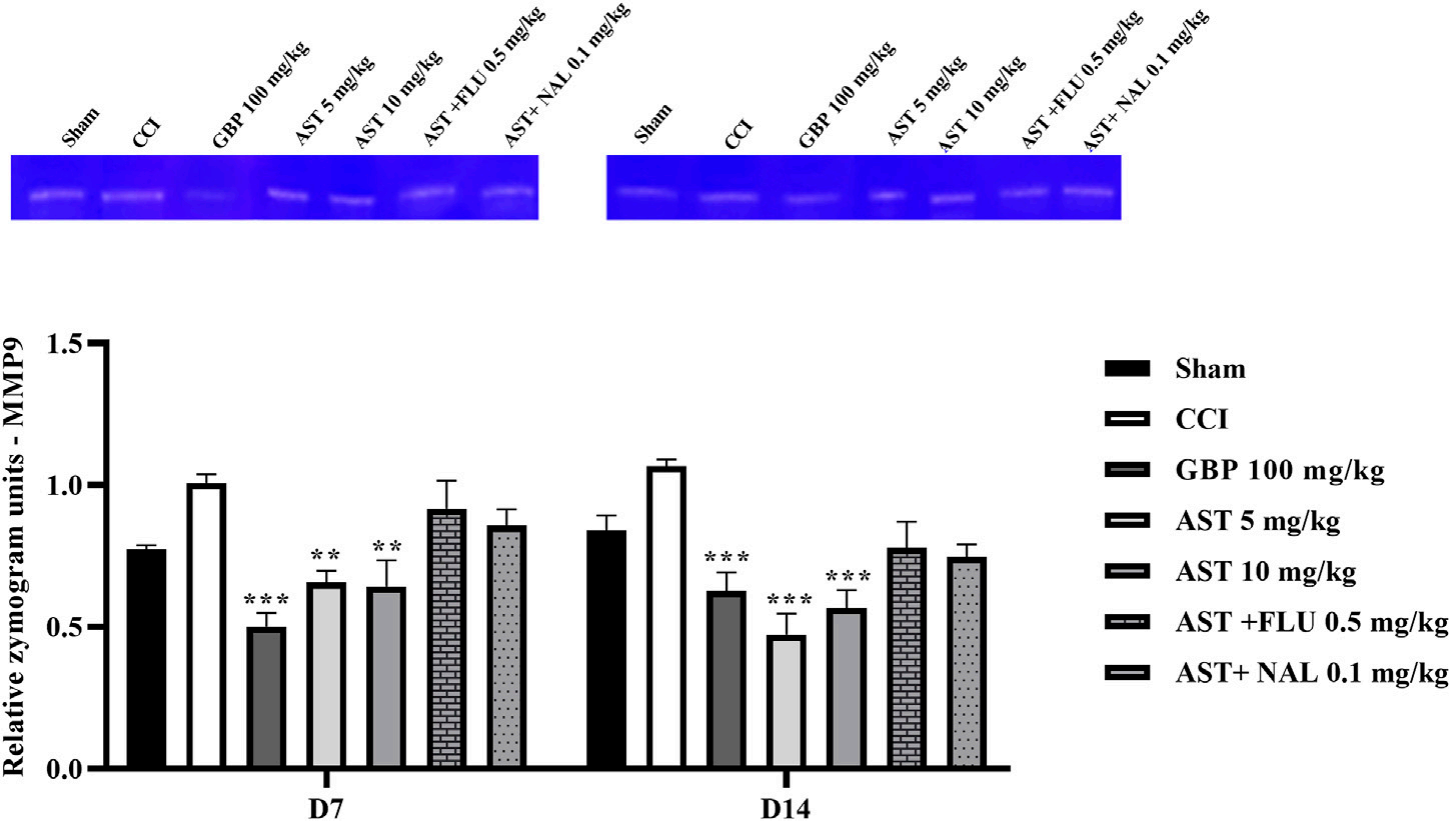
Effects of astaxanthin on serum MMP-9 activity after CCI in rats. The data are shown as mean ± SEM (*n* = 3). **p < 0.01, ***p < 0.001 vs. CCI. CCI, chronic constriction injury; GBP, Gabapentin; AST, Astaxanthin; FLU, Flumazenil; MMP, Matrix metalloproteinase; NAL, Naloxone.

The data showed an increase in the levels of MMP-9 (Figure 8) and MMP-2 (Figure 9) activity following CCI compared to the sham group (*p* < 0.001). Both doses of AST could reverse such increased patterns and had effects close to the effects of GBP (positive control) (*p* < 0.01). Injection of flumazenil and naloxone half an hour before AST administration reduced the positive effects of AST on MMP-2 and MMP-9 activity levels (*p* < 0.01).

### 3.8 Histopathological analysis

**FIGURE 10:**
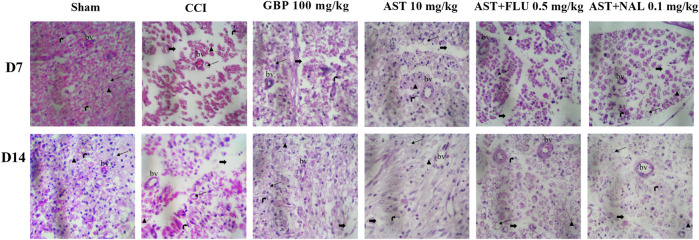
Representative photomicrographs (H&E, × 400) of sciatic sections from different groups following CCI in rats (*n* = 3). Thick black arrows indicate neuron gaps (wide separation between the nerve fibers), Thin black arrows indicate the area of axonal swelling, Schwann cell nucleus (right black arrow), Axon (arrow blackhead), bv: blood vessel. CCI, chronic constriction injury; GBP, Gabapentin; AST, Astaxanthin; FLU, Flumazenil; NAL, Naloxone.

In this study, we utilized H&E staining to assess tissue damage resulting from the CCI model and the impact of AST on it. The sciatic sections of the sham group exhibited well-organized myelin sheaths. Conversely, the stained nerve sections of the CCI group displayed axonal swelling, disrupted myelin sheaths, hemorrhage between cells, and wide separation between nerve fibers. However, CCI rats treated with AST (10 mg/kg) demonstrated a significant improvement in myelin sheath integrity and Schwann cells. These positive effects of AST significantly decreased in the AST + FLU (0.5 mg/kg) and AST + NAL (0.1 mg/kg) groups (Figure 10).

## 4 Discussion

**FIGURE 11:**
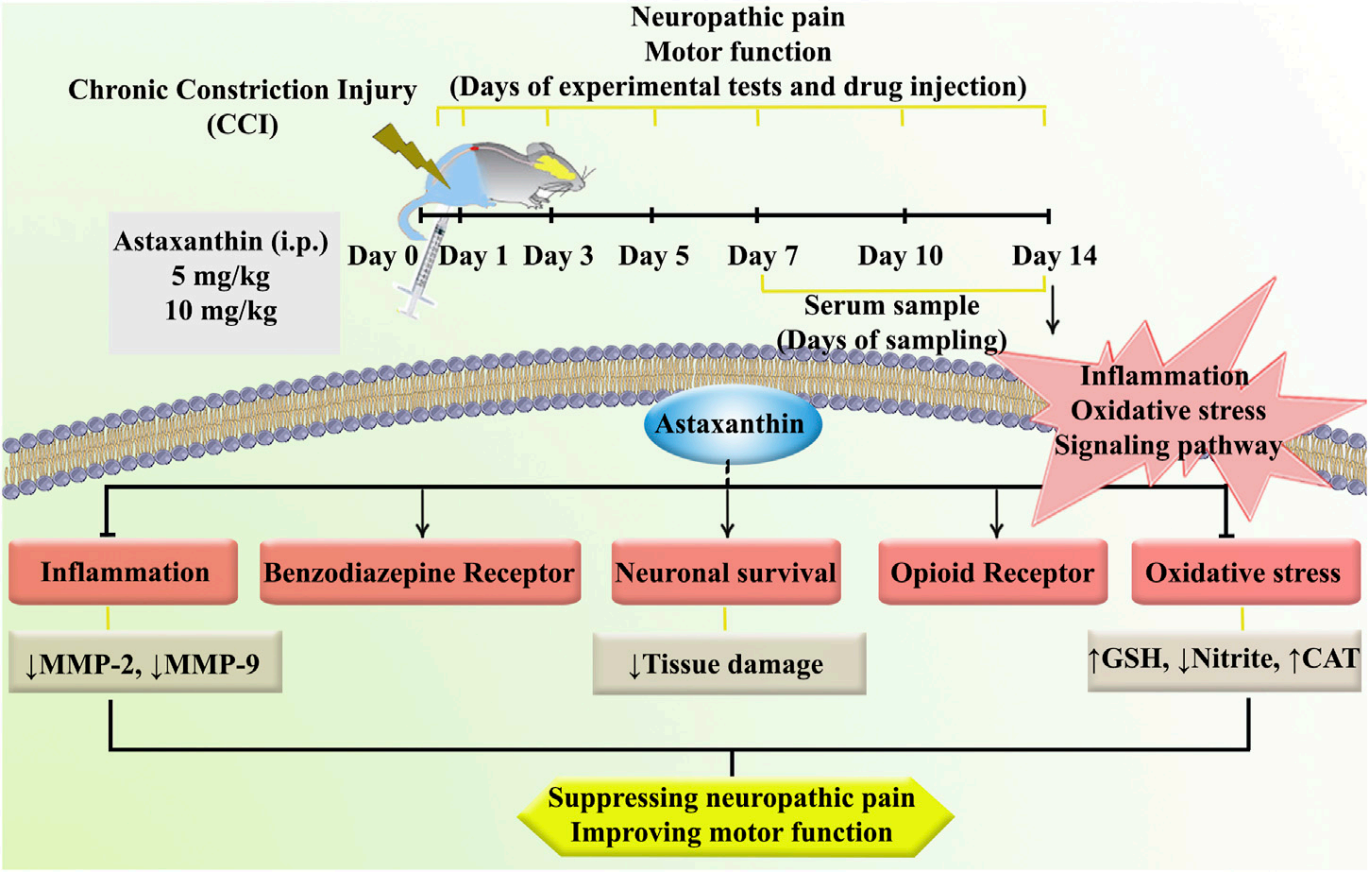
A summary of the research protocol and AST effects and mechanisms of action following CCI in rats.

The present study was designed to investigate the potential of using AST in a CCI model inducing neuropathic pain. Our study yielded several significant findings. Firstly, in rats with CCI, we observed a notable increase in heat hyperalgesia and cold allodynia, accompanied by a decline in motor performance over 14 days. Secondly, CCI rats exhibited elevated levels of MMP-9, MMP-2, and nitrite, while experiencing decreased levels of glutathione, and catalase activity, along with tissue alterations. Thirdly, treatment with AST demonstrated varying degrees of improvement in both behavioral and biochemical parameters. Lastly, the effects of AST appeared to be mediated through the benzodiazepine and opioid receptors, which are known to play roles in the management of neuropathic pain (Figure 11).

The CCI model is widely used to study neuropathic pain caused by nerve damage. This model effectively mimics the symptoms of neuropathic pain experienced by humans, such as allodynia and hyperalgesia, which are caused by peripheral nerve damage and inflammation. Additionally, compared to other peripheral neuropathy models, the CCI model induces long-lasting and widespread behavioral changes (Bennett and Xie, 1988; Austin et al., 2012). CCI-induced neuropathic pain is associated with the release of pro-inflammatory molecules, such as cytokines and chemokines, which activate immune cells and promote oxidative stress. One possible mechanism is the imbalance of the antioxidant defense system. Antioxidant enzymes like superoxide dismutase, glutathione peroxidase, and catalase are impaired, reducing the body’s ability to counteract oxidative stress (Naik et al., 2006). The second proposed mechanism is the activation of NADPH oxidase, an enzyme complex that generates ROS in peripheral nerves and the spinal cord following nerve injury. ROS can directly damage nerve tissues and increase the excitability of nociceptive neurons, leading to enhanced pain signaling (Cui et al., 2023). Additionally, nerve injury can disrupt mitochondrial function, leading to an increase in ROS production (Ilari et al., 2020). In line, other evidence also revealed a relationship between oxidative imbalance and neuropathic pain (Di Cesare Mannelli et al., 2016). Moreover, activation of pro-inflammatory pathways by the activation of glial cells and the release of inflammatory mediators, can induce oxidative stress and contribute to neuropathic pain development. This sustained neuroinflammation further contributes to neuropathic pain by promoting neuronal hyperexcitability and impairing nerve regeneration (Komirishetty et al., 2016; Ilari et al., 2020). Therefore, experimental interventions that can reduce oxidative stress can also reduce neuropathic pain symptoms in CCI models.

AST, a potent antioxidant found in marine organisms, has been proposed as a potential treatment for neuropathic pain due to its antioxidant, anti-inflammatory, and neuroprotective properties. Studies have shown that AST can improve behavioral and biochemical changes under *in vitro* and *in vivo* models of neuropathic pain. During a more related study with intraperitoneal administration of AST, we used 5, 10, and 20 mg/kg doses and found an inverted U-shaped dose-response effectiveness of AST against nociceptive pain (Mohammadi et al., 2021). Our previous results were in line with the quantitative features of the hormetic dose-response models (Calabrese, 2008). In the current study, in line with the findings of others, we showed that AST reduces neuropathic pain and improves motor performance by affecting oxidative stress specially at the dose of 10 mg/kg. This is achieved by the ability of AST to effectively inhibit MAPKs and nuclear factor-κB pathways, mitigate oxidative stress, and suppress the production of inflammatory cytokines (Zhao et al., 2021; Ciapała et al., 2023).

On the other hand, the upregulation of MMP-9 and MMP-2 in the peripheral nerves contributes to the development and maintenance of neuropathic pain after CCI. MMP-9 and MMP-2 activate pro-inflammatory pathways such as the release of pro-inflammatory cytokines and chemokines. Such inflammatory mediators can further perpetuate the inflammatory response and contribute to the development and maintenance of neuropathic pain. They can also modulate the activity of ion channels and receptors involved in pain signaling, such as N-methyl-D-aspartate receptors and transient receptor potential channels, leading to increased pain sensitivity. MMP-9 induces early-phase neuropathic pain by activating interleukin (IL)-1β and microglia in the early phase, while MMP-2 is implicated in the development of late-phase neuropathic pain. Therefore, inhibition of MMP-9 or MMP-2 may help reduce neuropathic pain (Ru-Rong et al., 2008; Lakhan and Avramut, 2012; Gu et al., 2019). In a study by Ciapała et al. (2023), the analgesic effect of a single intrathecal dose of AST in mice during 7 days was shown to be mediated through inhibiting mitogen-activated protein kinase and activating nuclear factor erythroid 2–related factor 2. They employed von Frey and cold plate tests for behavioral analysis and found that intrathecal administration of AST with morphine, buprenorphine or oxycodone resulted in greater decreases in tactile and cold allodynia. In our study and in comparison, for the first time we evaluated the anti-neuropathic effects of intraperitoneal AST in rats during 14 days. Additionally, we employed more behavioral tests, including von Frey, hot plate, acetone drop, as well as the motor-related test of open field to assess rearing, crossing, and grooming. From a mechanistic aspect, possible involvement of opioid and benzodiazepine receptors in the anti-neuropathic effects of AST was examined using the corresponding antagonists, naloxone and flumazenil, respectively. From a molecular point of view, we evaluated oxidative (i.e., nitrite) and antioxidant (i.e., CAT and GSH) mediators, as well as inflammatory ones (e.g., MMP2 and MMP9). Also, in our study histopathological changes were compared in different groups (Ciapała et al., 2023).

Both benzodiazepine receptors (specifically the GABA-A receptor) and opioid receptors (such as mu, delta, and kappa receptors) play roles in pain modulation. They can interact with each other and have synergistic effects in reducing pain perception. Activation of benzodiazepine receptors enhances the analgesic effects of opioids, and vice versa (Gear et al., 1997; Primeaux et al., 2006). CCI has been found to affect the expression and function of benzodiazepine and opioid receptors, thereby impacting inhibitory neurotransmission and playing a role in the modulation of pain processing. Specifically, CCI leads to a decrease in benzodiazepine receptor binding, potentially contributing to the manifestation of anxiety-like behaviors (Shih et al., 2008; Lewis et al., 2012). We also found that AST successfully prevented hyperalgesia and anxiety-like behaviors in the CCI model. Furthermore, when we administered the benzodiazepine antagonist flumazenil or naloxone with AST, the therapeutic effect of AST was suppressed.

According to our tissue analysis, the effect of AST in reducing neuropathic pain is associated with maintaining the structural integrity of the sciatic nerve, regulating myelin sheaths, hemorrhage between cells, and wide separation between nerve fibers. Suppressing inflammation and oxidative stress could lead to tissue repair during 14 days of AST treatment, which passes through benzodiazepine/opioid receptors. Blocking opioid and benzodiazepine receptors also reduced the therapeutic potential of AST and tissue repair.

## 5 Conclusion

In conclusion, the findings demonstrated the promising potential of AST as a viable candidate for neuropathic pain treatment and motor function. Through its ability to pass through opioid/benzodiazepine receptors, AST has the potential to suppress inflammatory and oxidative mediators, while enhance antioxidant factors. Further research and clinical trials are warranted to solidify its efficacy and safety profile.

## Data Availability

The datasets generated during and/or analyzed during the current study are available from the corresponding author upon reasonable request.

## Supplementary material

SUPPLEMENTARY FIGURE 1 Refusing the role of intraperitoneal solvent volume on neuropathic pain after CCI. No difference was shown between sham and sham + v2 and also between CCI and CCI + v2 in different days. The data are shown as mean ± SEM (*n* = 6). ^+++^ *p* < 0.01 vs. sham, ****p* < 0.001 vs. sham + v2. CCI: chronic constriction injury, DW: distilled water. Sham [Sham receiving DMSO 5% (v1)], Sham + v2 [Sham receiving DMSO 5% (v1) + DW (v2)], CCI [CCI receiving DMSO 5% (v1)], CCI + v2 [CCI receiving DMSO 5% (v1) + DW (v2)].

SUPPLEMENTARY FIGURE 2 Refusing the role of intraperitoneal solvent volume on motor dysfunction after CCI. No difference was shown between sham and sham + v2 and also between CCI and CCI + v2 in different days. The data are shown as mean ± SEM (*n* = 6). ^+++^ *p* < 0.01 vs. sham, ****p* < 0.001 vs. sham+v2. CCI: chronic constriction injury, DW: distilled water. Sham [Sham receiving DMSO 5% (v1)], Sham + v2 [Sham receiving DMSO 5% (v1) + DW (v2)], CCI [CCI receiving DMSO 5% (v1)], CCI + v2 [CCI receiving DMSO 5% (v1) + DW (v2)].

## Abbreviations

AST, astaxanthin; AUC, area under the curve; CCI, chronic constriction injury; DMSO, dimethylsulfoxide; DTNB, 5,5′-dithio-bis(2-nitrobenzoic acid); FLU, flumazenil; H_2_O_2_, hydrogen peroxide; MMP, matrix metalloproteinase; NAL, naloxone; NEDD, diamine dihydrochloride; OD, optical density; PWL, paw withdrawal; ROS, reactive oxygen species.
