# Supplementary figures and images for: Anti-neuropathic effects of astaxanthin in a rat model of chronic constriction injury: passing through opioid/benzodiazepine receptors and relevance to its antioxidant and anti-inflammatory effects
Source: Front Pharmacol. 2024 Nov 25;15:1467788. doi: 10.3389/fphar.2024.1467788 (PMC11625551; doi:10.3389/fphar.2024.1467788)

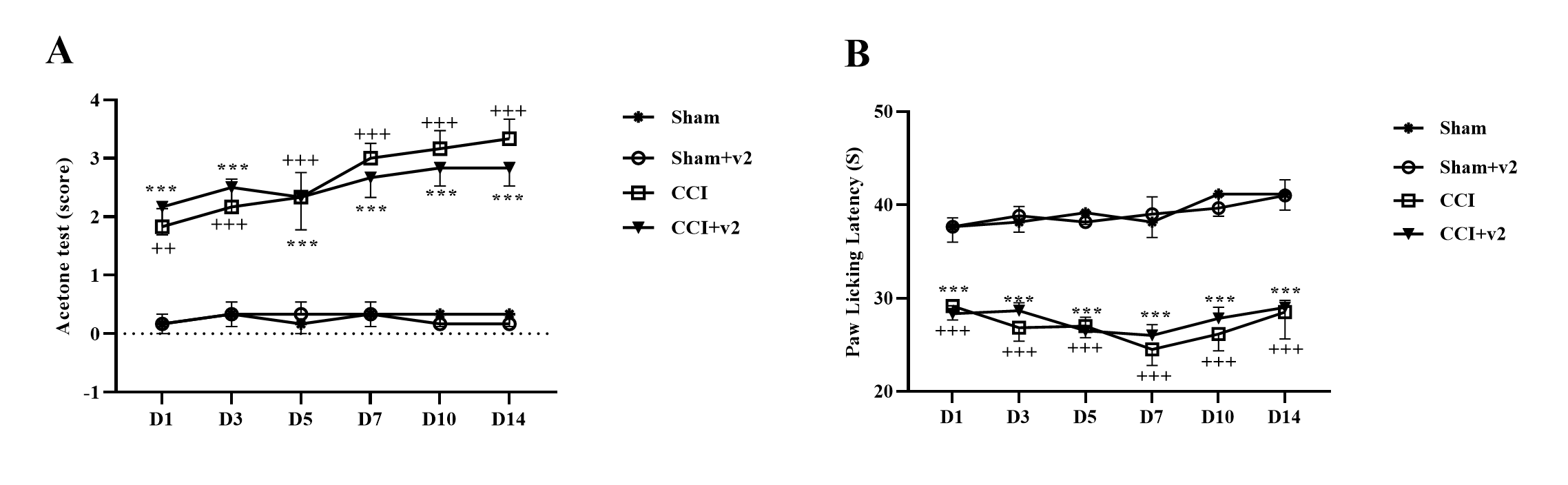

Supplement: Supplementary file 1 [file Image2.TIF]

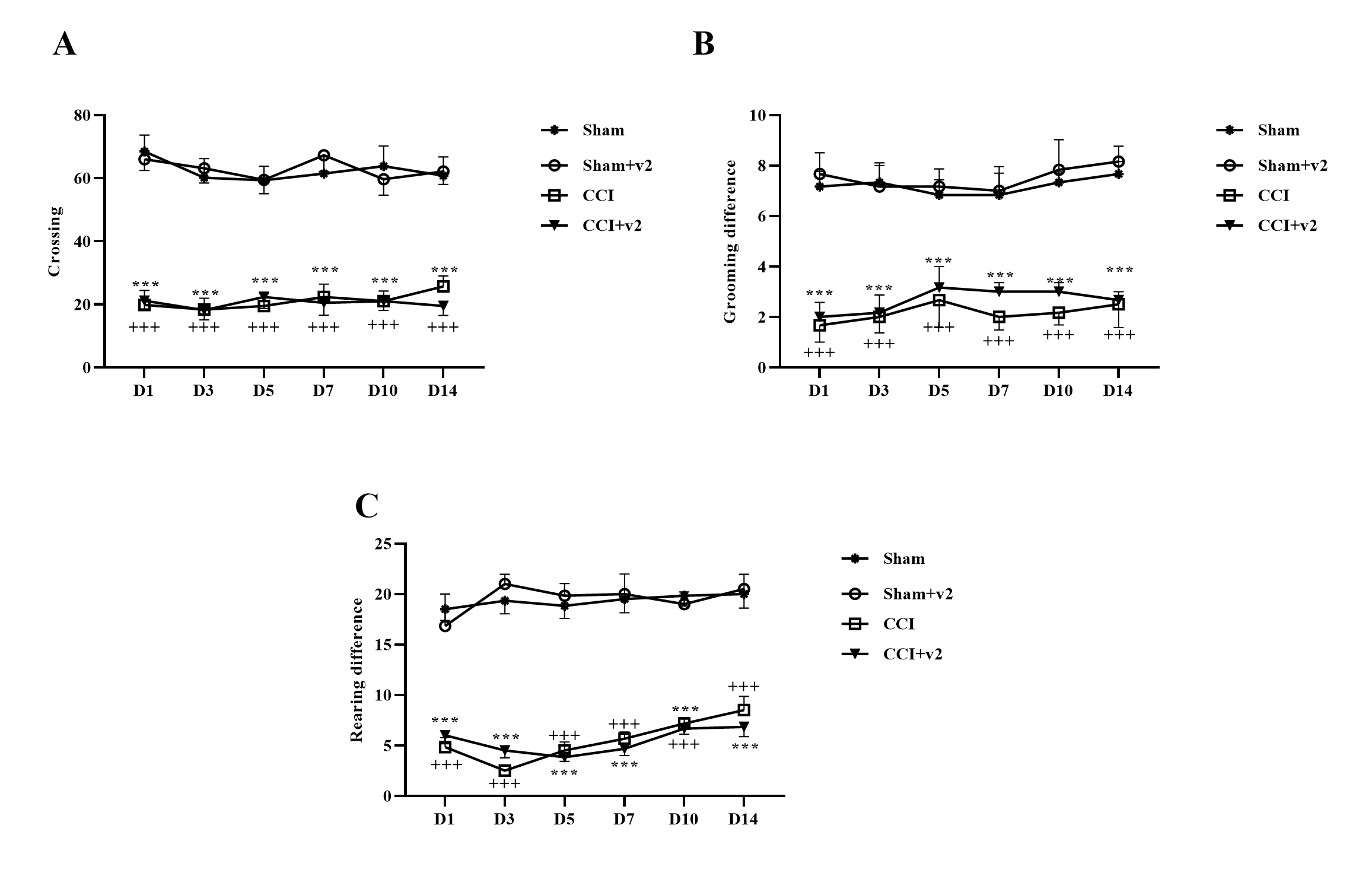

Supplement: Supplementary file 2 [file Image1.TIF]
